# Sneezing

**Published:** 1858-04

**Authors:** 


### SNEEZING

Is rather a pleasant operation than otherwise, when a person is alone. But there are inopportune sneezes, to wit: for a young man to sneeze just at the moment of popping the question; or, for it to accompany the lady-love's "yes!" Besides, many persons are not to be sneezed at. Inasmuch, therefore, as a sneeze is most tremendously out of place; sometimes, it is well enough to know how it may be prevented.

A sneeze is instantaneously dispersed, dispelled, scouted, broken up, by pressing the finger upwards against the division of the nose, at the point where the upper-lip, inside, joins the gum.

Another plan is, to expire all the air possible from the lungs, the moment you perceive indications of a sneeze.

